# Osteoporosis Is Associated with Deteriorating Clinical Status in Adults with Cystic Fibrosis

**DOI:** 10.1155/2018/4803974

**Published:** 2018-03-26

**Authors:** Inger Hee Mathiesen, Tacjana Pressler, Peter Oturai, Terese Lea Katzenstein, Marianne Skov, Ruth Frikke-Schmidt, Mette Friberg Hitz

**Affiliations:** ^1^Department of Infectious Diseases, Copenhagen Cystic Fibrosis Center, Rigshospitalet, Blegdamsvej 9, 2100 Copenhagen, Denmark; ^2^Danish Pediatric Pulmonary Service, Copenhagen Cystic Fibrosis Center, Rigshospitalet, Blegdamsvej 9, 2100 Copenhagen, Denmark; ^3^Department of Clinical Physiology, Nuclear Medicine & PET, Rigshospitalet, Blegdamsvej 9, 2100 Copenhagen, Denmark; ^4^Department of Clinical Biochemistry, Rigshospitalet, Blegdamsvej 9, 2100 Copenhagen, Denmark; ^5^Department of Medicine, Endocrine Division, Zealand University Hospital, Lykkebaekvej 1, 4600 Koege, Denmark

## Abstract

**Background:**

Cystic fibrosis (CF) patients are in increased risk of osteoporosis. We aimed to determine the osteoporosis prevalence in an adult CF cohort and investigate calcium metabolic parameters and clinical status' association with bone mineral density evaluated by dual X-ray absorptiometry scan.

**Methods:**

We performed a cross section database study of adults at a tertiary CF Center. *Z* scores were applied for patients < 50 years of age and *T* scores for patients > 50 years of age.

**Results:**

One hundred twenty-five patients were included. Compared to nonosteoporotic patients, osteoporotic patients (15%) had significantly lower percent predicted forced expiratory volume in 1 second (ppFEV1), lower body mass index, higher frequency of CF-related diabetes and chronic lung infection, and higher high-sensitive C-reactive protein and glycated hemoglobin levels. Vitamin D was not associated with any outcome. In multivariate analyses, only ppFEV1 and female gender were independently associated with *Z* scores.

**Conclusions:**

Osteoporosis in CF occurs with deteriorating clinical status while the role of calcium metabolism seems minor. Gender specific and dysglycemic impact on bone status should be investigated further.

## 1. Introduction

As treatments for cystic fibrosis have improved, the median life expectancy has increased over the last decades [1]. However, as lifespan increases so does the risk of comorbidities. One of these is osteoporosis, and CF patients have increased risk of osteoporosis compared to age-matched controls [2, 3]. Another frequent feature of CF is vitamin D deficiency [2, 3].

A silent disease, osteoporosis has no clinical manifestations but the consequence—osteoporotic fractures—does. Osteoporotic fractures may have severe consequences for CF patients. Low-impact fractures, for example, costa fractures from coughing may worsen their pulmonary condition as reduced coughing, due to thoracic pains, will lead to sputum stagnation and reduced airway clearance. This will favor the conditions for bacterial growth, ultimately leading to pulmonary exacerbation [4]. Furthermore, vertebral fractures can lead to anatomic changes of the thorax, which may impede or even rule out lung transplantation [2, 3, 5, 6].

Factors such as calcium metabolic alterations, malabsorption, delayed puberty, chronic inflammation, and many other conditions associated with CF [2, 7, 8] can prevent patients from achieving peak bone mass equal to their healthy peers and increase bone loss in adulthood.

The aim of the study was to determine the prevalence of osteoporosis in a cohort of well-treated CF adults and investigate clinical and calcium metabolic parameter's association with BMD.

## 2. Methods

We performed a cross section study of adults at the Copenhagen CF Center.

All CF patients are followed monthly with assessment of clinical status (lung function parameters, body mass index (BMI), and sputum microbiology) at the CF outpatient clinic. An annual review is performed including extensive biochemical evaluation. Clinical data and prescribed medications are prospectively registered in patient records and the CF Center database.

Patients are routinely supplemented with fat-soluble vitamins A, D (D_3_) (2400 International Units (IU) cholecalciferol), E, and K.

Study inclusion criteria were patients with a confirmed CF diagnosis by genotyping and/or sweat test aged ≥20 years attending the Copenhagen CF Center regularly (at least 4 times a year), with at least one DXA scan between 2010 and 2015. Patients with a history of solid organ transplantation were excluded.

The following data were extracted from the CF database: weight, height, BMI (average BMI within the same year as DXA and blood samples were performed), CF genotype, gender, and age. Further, information about chronic bacterial lung infection, pancreas insufficiency, CF-related diabetes (CFRD), and lung function (ppFEV1) [9, 10] were obtained. From medical records, information regarding additional vitamin D_3_ beyond routine supplementation, contraceptives, inhaled steroids, PPI use, and bone antiresorptive treatment (bisphosphonates) were obtained.

### 2.1. Bone Densitometry

Bone mineral density (BMD) was determined with DXA scan (Lunar Prodigy Pro, GE Healthcare, Madison, WI, USA). BMD was measured in the lumbar spine (L2–L4) and in the right and left femoral neck and trochanter regions. All measurements were performed by trained technicians and reviewed by trained physicians, and the same scanner was used for all measurements. Results were expressed as BMD (g/cm^2^), *T* scores (the number of standard deviations a given BMD value differs from the mean value of young healthy adults), and *Z* scores (the number of standard deviations a given BMD value differs from the mean of healthy age- and gender-matched controls). Scores were calculated with a German normal reference material provided by the manufacturer. International Society for Clinical Densitometry (ISCD) criteria for younger males < 50 years of age and premenopausal females were applied, defining *Z* scores < −2 as BMD below expected range for age and *Z* scores ≥ −2 as BMD within expected range for age [11], which was then defined as osteoporosis and BMD within the normal range, respectively. Participants above 50 years of age were grouped according to WHO criteria definitions [12] with *T* scores ≤ −2.5 defining osteoporosis. Left femur was used in analyses unless otherwise stated.

### 2.2. Blood Tests

From an extended blood panel drawn the same year as DXA scan was performed, the following parameters were available: vitamin D (25OHD) (vitamin D_3_ + vitamin D_2_), alkaline liver transaminase (ALT), alkaline phosphatase, hemoglobin A1c (hbA1c), creatinine, thyroid-stimulating hormone (TSH), total immunoglobulin G (IgG), albumin, magnesium (Mg^2+^), and phosphate (PH^−^). Follicle-stimulating hormone (FSH), luteinizing hormone (LH), total testosterone, total estradiol, parathyroid hormone (PTH), total calcium, and high-sensitive C-reactive protein (hsCRP) were analyzed from frozen serum samples collected at the same sample date as the extended blood panel. All frozen samples were stored at −80°C until analysis. Total testosterone, total estradiol, FSH, LH, and PTH were measured by electrochemiluminescence immunoassays (ECLIA) (Cobas, Roche, Switzerland). Coefficient of variation for these was PTH 7%, FSH and LH 7%, total testosterone 6% and 5% at levels 3 nmol/l and 35 nmol/l, respectively, and total estradiol 11% and 7% at levels 0.2 nmol/l and 2.5 nmol/l, respectively.

### 2.3. Lung Function Test

Lung function tests were performed at every outpatient visit according to American Thoracic Society/European Respiratory Society (ATS/ERS) guidelines [9, 10] using Jaeger Master Screen Pro (CareFusion, Hochberg, Germany). For the study, mean ppFEV1 of all measurements within the year of DXA scan was calculated and used.

### 2.4. Statistical Analysis

Descriptive statistics are presented as median and interquartile range (IQR), mean and standard deviation (SD) or percentage (%) as appropriate. ANOVA, parametric/nonparametric *T*-test according to distribution, or chi-square test were used to compare groups (vitamin D levels, men versus women, normal BMD versus osteoporosis). When groups were very unequal in sample size, Welch *T*-test was used. Univariate regression analysis was performed to evaluate associations between bone measurements and factors. Nonnormal distributed data were log10 transformed or square rooted. Finally, a predefined multivariate linear regression (MLR) model was performed for both *T* and *Z* scores containing the variables gender, ppFEV1, BMI, chronic infection (no/yes), hsCRP, 25OHD, and estradiol. For regression analyses, both *Z* and *T* scores were used as much previous literature in adults is based on *T* scores. All analyses were performed with SPSS ver. 22. A *p* value < 0.05 was considered statistically significant.

The study protocol was approved by the Local Ethics Committee (H-15008060) and Local Data Protection Agency (RH-2015-186, I-suite number 04107).

## 3. Results

Of 172 nonlung transplanted patients ≥ 20 years of age, 125 patients fulfilled the inclusion criteria and were included.

Baseline characteristics are depicted in Table 1. Ten patients were scanned in 2012, 31 patients in 2013, 53 patients in 2014, and 31 patients in 2015.

## 4. Vitamin D

**Figure 1:**
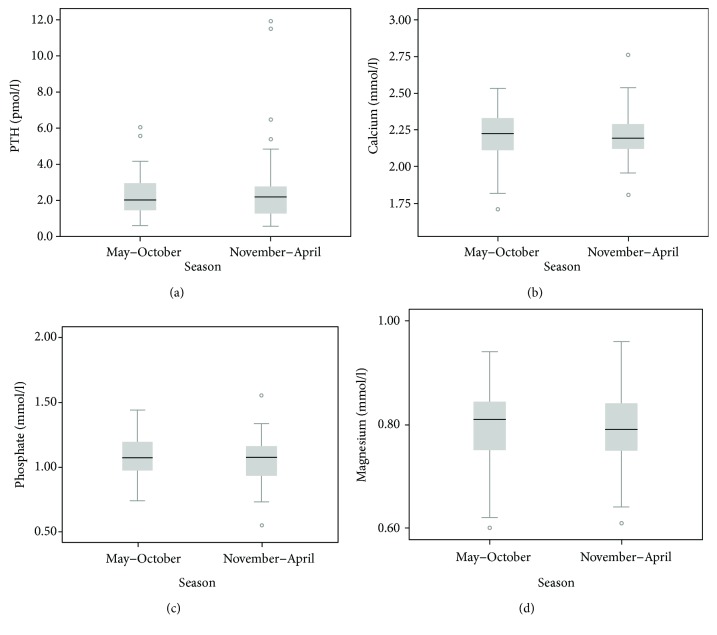
Seasonal variation in calcium metabolic parameters.

The median (IQR) 25OHD level in the cohort was 49.5 nmol/l (32–71) (Table 2). Twenty-four patients (19%) had 25OHD levels ≥ 75 nmol/l. Of those, twelve (50%) received additional vitamin D_3_ (mean 400 IU daily, IQR: 400 IU–800 IU) in addition to routine supplementation of 2400 IU daily. In total, forty seven patients were prescribed additional D_3_ substitution besides routine supplementation but there was no significant difference in 25OHD levels between receivers and nonreceivers of extra D_3_, mean levels 51 nmol/l and 53 nmol/l, *p* = 0.7, respectively. Overall, the 25OHD level was significantly higher in patients, who had their 25OHD levels measured during May–October than during the winter months (mean levels 59.6 nmol/l versus 47.1 nmol/l, *p* = 0.02). There was no seasonal variation in other calcium metabolic parameters, that is, PTH, total calcium, Mg^2+^, or PH^−^ (Figure 1).

Patients were subgrouped according to 25OHD levels as deficient < 25 nmol/l, insufficient > 25–50 nmol/l, and replete > 50 nmol/l. There was no statistical significant difference in baseline characteristics. Parathyroid hormone was significantly higher in the deficiency group compared to the insufficient group (mean difference 1.35, 95% CI 0.27–2.43, *p* = 0.006) and replete group (mean difference 1.43, 95% CI 0.52–2.34, *p* = 0.001). Total calcium levels did not differ between groups, but PH^−^ was significantly lower in the deficiency group compared to the replete group (mean difference between groups 0.13, 95% CI: 0.03–0.23, *p* = 0.005). However, both mean PTH values and mean PH^−^ values were within normal range in all vitamin D groups (Table 3). Neither Mg^2+^ nor alkaline phosphatase was significantly different between groups.

### 4.1. Hormones

Hormones were analyzed gender specific.

Amongst women, it was not possible to retrieve data regarding menstrual cycle, possible amenorrhea, or peri/postmenopausal stage. Six women were according to medical records taking oral contraceptives and excluded from further hormonal analyses. Hormonal levels are depicted in Table 4. The majority of women below the age of 50 had estradiol levels below 0.5 nmol/l.

In men, median estradiol level was 0.1 nmol/l (0.09–0.23). Overall median testosterone for men < 50 years of age was 2.6 (0.73–13.46).

### 4.2. Bone Status Assessment

The overall DXA scan results are depicted in Table 1. Nineteen patients (15%) had osteoporosis, fifteen patients (12%) had *Z* scores below the expected range for age (*Z* scores < −2), and four patients had *T* scores ≤ −2.5 and were above 50 years of age. Mean *Z* scores of the overall cohort were significantly lower compared to the mean healthy age-matched general population and mean difference between groups was −0.748 (95% CI: −0.95 to −0.55), *p* < 0.001. Patients homozygous for the F508del had significantly lower BMD than nonhomozygous patients. For femoral BMD, mean difference between groups was −0.09 (95% CI: −0.16 to −0.01), *p* = 0.03, and for lumbar BMD, mean difference between groups was −0.07 (95% CI: −0.13 to −0.01), *p* = 0.02.

Compared to the group with BMD values within the normal range, osteoporotic patients had significantly lower average ppFEV1 (*p* < 0.001), BMI (*p* < 0.001), and albumin (*p* = 0.01), but there was no significant difference in age (34 years versus 31 years, *p* = 0.19) or gender distribution in groups (*p* = 0.16) (Table 5).

The osteoporosis group was more frequently chronically infected (*p* = 0.02) and had higher hsCRP levels (*p* = 0.04), but there was no difference in IgG between groups.

Patients with osteoporosis also had a higher frequency (*p* = 0.03) but not a longer duration of CFRD (*p* = 0.06), and hbA1c was significantly higher compared to the group with BMD values within the normal range (*p* < 0.001).

ALT was higher among patients with osteoporosis, but no other calcium metabolic-, liver-, and kidney parameters differed between the groups (Table 2).

There was no difference in frequency of PPI or steroid IH usage between groups (Table 5).

Estradiol levels were significantly lower in osteoporotic women compared to women with BMD within the normal range (Table 4). In men, median estradiol levels were at the lower limit of the reference interval (0.09 nmol/l) for both groups; no sex hormone levels differed between osteoporotic and nonosteoporotic men.

### 4.3. Regression Analyses

Univariate associations between bone status and clinical and paraclinical parameters are presented in Table 6. In multivariate regression analysis (MLR), femoral *Z* and *T* scores were independently associated with ppFEV1 and chronic infection. Spine *Z* and *T* scores were independently associated with ppFEV1, chronic infection, and female gender, and spine *T* score was also with BMI (Table 7).

## 5. Discussion

In a well-treated and closely monitored cohort of adult CF patients, we found that 15% had osteoporosis, which is comparable to other CF cohorts [5]. *Z* scores were significantly lower compared to a healthy, age-matched population, and bone density was independently associated with ppFEV1, chronic infection, and female gender.

Consistent with previous findings, ppFEV1 was significantly associated with BMD [5, 6, 13, 14]. As well, BMI and albumin were significantly lower while CFRD and chronic infection frequencies were significantly higher in osteoporotic patients compared to patients with BMD values within the normal range. And patients homozygous for the F508del mutation had significant lower BMD compared to patients bearing only one or no F508del mutation. These findings all represent disease severity and progression confirming that clinical status is an important factor for impaired bone strength in CF [13–16]. The fact that mean *Z* score was significantly below the average healthy mean also justifies more frequent monitoring of BMD in CF populations in order to identify patients at increased risk.

Cross-sectional studies in CF and other respiratory diseases [17, 18] have observed findings similar to ours between decreased BMD and lung function, but it is not yet determined, whether it is the decreasing lung function itself or other factors induced by declining FEV1, which mediates the decreased BMD. In CF, a suspected link between lung function and decreasing BMD is systemic inflammation. Studies have described associations between pulmonary exacerbations, increased osteoclast maturation and activity [19, 20], and increased levels of inflammatory cytokines known to promote bone resorption, for example, IL-6, IL-1, and TNF*α* [21, 22]. The significant and independent association of chronic infection with femoral *Z* score in our study and the higher hsCRP levels in osteoporotic patients support the relation between inflammation and bone loss.

The median 25OHD levels were considerably lower than Endocrine Society [23] and CFF [24] recommendations, and contrary to established pathogenetic understanding of decreased bone strength, there was no difference in 25OHD across between osteoporotic and nonosteoporotic groups. Noteworthy is the difference between summer and winter levels indicating that exposure to UVB radiation may be an effective way of increasing vitamin D levels bypassing malabsorption from the gut. The seasonal variation is also noted in other CF studies [25, 26] and may explain why it is difficult to determine a link between BMD and 25OHD levels. CF patients though, especially when chronically infected and deteriorating in lung function, are subject to a heavy treatment burden, and several studies of adherence to therapy in CF have consistently shown that therapy compliance often is as low as 50% of prescribed medications [27, 28]; this may further obscure a relationship between prescribed vitamin D doses and blood levels measured.

After all, our—as well as others'—findings [29–31] indicate that the role of vitamin D in CF bone disease may be minor compared to other complications negatively affecting bone mass such as clinical status and possibly inflammatory bone loss.

That said, intervention studies investigating bone status longitudinally have used inadequate ergocalciferol (D_2_)/cholecalciferol (D_3_) doses of less than 3000 IU/day [31], while at present, there is consensus that oral doses of 50,000 IU D_3_ weekly for at least eight weeks are likely needed to significantly increase 25OHD levels above 75 nmol/l in vitamin D-deficient CF patients [8, 23, 32].

Interestingly, osteoporotic patients had higher prevalence of CFRD and poorer glycemic control (hbA1c) compared to patients with normal BMD, and there was a trend towards longer duration of CFRD in the osteoporotic group. There might be several ways in which CFRD contributes to CF bone disease. A recent study demonstrated elevated levels of receptor of advanced glycation end products (RAGE) and advanced glycation end products (AGE) in CF patients with CFRD compared to CF patients without CFRD and healthy controls [33]. While RAGE can enhance bone resorption through stimulation of proinflammatory cytokines and oxidative stress species [34], AGE cross-linking in bone could render CF bones more susceptible to fracture [35]. Further, osteoblast function/formation could be affected [36], as insulinopenia, which is present in CFRD, has been shown to cause impaired fracture healing [37] and bone formation deficits [38] in rodent models. The importance of these mechanisms in CF bone disease is yet to be determined.

Male patients had completely suppressed estradiol levels in both the osteoporotic and nonosteoporotic group despite estradiol being the most important sex hormone for bone in males as well as females [39]. The seemingly low testosterone levels point to male hypogonadism, which in conjunction with the completely suppressed estradiol, indicate possible sex hormone alterations, which should be investigated further together with its possible impact on BMD in CF males.

We acknowledge that there are limitations to our study. We did not have reliable information about oral/intravenous corticosteroid use which would have allowed evaluation of systemic steroids on BMD in the cohort. However, these are generally only used for intermittent worsening of CF arthritis or treatment of allergic bronchopulmonary aspergillosis. Hypothetically, though, such repeated high-dose prednisone in adolescence could have a negative impact on peak bone mass accrual making a subgroup more susceptible to loss of bone mass in adulthood.

Neither was it possible to measure free fractions of sex hormones, obtain history of menstrual cycle or symptoms of hypogonadism from patient records, preventing any final conclusions about sex hormones' impact on CF bone status. Finally, we did not have any radiographic data or patient history to determine correlation between any osteoporotic fractures and BMD, a relationship which is being questioned over the recent years as peripheral quantitative computed tomography scans have shown altered microarchitecture in CF bone independently of BMD [40].

In conclusion, CF patients develop impaired BMD associated with deteriorating clinical status. It is therefore important to identify these individuals at risk and ensure regular DXA scans and optimize treatment of modifiable risk factors of osteoporosis. Although the skeleton is the body's calcium storage, there was little indication that alterations in calcium metabolism significantly impact bone status, despite median vitamin D levels being well below recommended values. Long-term high-dose vitamin D studies are needed to clarify the role of 25OHD in CF bone disease and to which extent it may have an effect on BMD longitudinally. Sex hormonal and dysglycemic impact on bone status should be investigated further.

## Figures and Tables

**Table 1:** Baseline characteristics.

	Overall
ppFEV1 (SD)	67 ± 23
BMI kg/m^2^ (SD)	22.3 ± 3.5
Age, years (SD)	33.1 ± 10
Males, *n* (%)	62 (50.4)
Females, *n* (%)	63 (49.6)
Pancreas insufficiency, *n* (%)	120 (97)
Homozygous F508del, *n* (%)	89 (71)
CF-related diabetes, *n* (%)	51 (41)
CF-related diabetes, duration in years (IQR)	10 (6–18)
Chronic infection, *n* (%)	101 (81)
Steroid inhalation therapy, *n* (%)	55 (44)
Additional D_3_ supplementation, *n* (%)	47 (38)
Proton pump inhibitors, *n* (%)	44 (35)
Spine BMD	1.145 ± 0.158
Femoral BMD	0.974 ± 0.197
*T* score	−0.981 ± 1.260
*Z* score	−0.748 ± 1.110

ppFEV1: percent predicted forced expiratory volume in 1 second; BMI: body mass index; extra D_3_ supplementation: additional D_3_ supplementation on top of routine supplementation; BMD: bone mineral density; *T* score: lowest *T* score at any site for cases; *Z* score: lowest *Z* score at any site for cases; IQR: interquartile range; SD: standard deviation.

**Table 2:** Calcium metabolic and biochemical differences between groups.

	Normal range	Overall	BMD within the normal range	Osteoporosis	*p* between groups
25OHD (*n* = 121)	(>50 nmol/l)	49 (32–71)	49 (34–71)	32 (16–71)	0.39
Calcium (*n* = 125)	(2.15–2.51 mmol/l)	2.21 (2.12–2.31)	2.22 (2.13–2.32)	2.12 (2.02–2.28)	0.26
PTH (*n* = 125)	(1.6–6.9 pmol/l)	2.12 (1.36–2.91)	2.12 (1.37–2.76)	2.13 (1.6–4.86)	0.18
Mg^2+^ (*n* = 117)	(0.71–0.94 mmol/l)	0.8 (0.75–0.84)	0.8 (0.76–0.84)	0.77 (0.7–0.87)	0.18
PH^−^ (*n* = 117)	(0.71–1.53 mmol/l)	1.07 (0.96–1.18)	1.07 (0.96–1.16)	1.11 (0.94–1.29)	0.44
A phosphatase (*n* = 123)	(35–105 U/l)	94 (77–122)	89 (76–120)	112 (83–170)	0.11
Zink (*n* = 110)	(10–19 *μ*mol/l)	13 (11–14)	13 (11–15)	11 (9–13)	0.13
Albumin (*N* = 122)	(36–48 g/l)	38 (34–42)	39 (34–42)	37 (30–39)	0.02
IgG (*n* = 121)	(6.1–14.9 g/l)	11.2 (9–13.7)	11.2 (9.00–13.25)	12.00 (8.4–14.90)	0.46
hsCRP (*n* = 125)	(<3.00 mg/l)	2.34 (1.00–6.97)	2.10 (0.93–6.94)	4.52 (2.80–8.22)	0.04
Urea (*n* = 124)	(3.2–8.1 mmol/l)	4.7 (3.93–6.18)	4.70 (3.90–6.10)	5.00 (4.20–6.80)	0.84
Creatinine (*n* = 124)	(60–105 *μ*mol/l)	70 (60–83)	69 (60–81)	73 (63–104)	0.13
ALT (*n* = 123)	(10–70 U/l)	26 (19–39)	26 (19–42)	28 (19–31)	0.02
LDH (*n* = 122)	(115–255 U/l)	158 (142–176)	159 (142–179)	152 (140–170)	0.43
hbA1c (*n* = 122)	(<48 mmol/mol)	41 (36–53)	40 (36–49)	55 (40–78)	0.007
TSH (*n* = 122)	(0.65–4.80 × 10^−3^ IU/l)	1.85 (1.24–2.58)	1.87 (1.25–2.61)	1.81 (0.92–2.56)	0.90

All data presented as median and interquartile range (IQR). 25OHD: vitamin D; PTH: parathyroid hormone; Mg^2+^: magnesium; PH^−^: phosphate; A phosphatase: alkaline phosphatase; IgG: immunoglobulin G; hsCRP: high-sensitive C-reactive protein; ALT: alanine aminotransferase; LDH: lactate dehydrogenase; hbA1c: hemoglobin A1c; TSH: thyroid-stimulating hormone; m: milli; *μ*: micro; n: nano; pmol: picomol; l: liter; IU: international units; g: gram.

**Table 3:** Calcium metabolic parameters by vitamin D status.

	Normal range	Deficient	Insufficient	Replete	*p*
Calcium	(2.15–2.51 mmol/l)	2.17 (0.16)	2.21 (0.14)	2.23 (0.17)	0.27
Phosphate	(0.71–1.53 mmol/l)	1.16 (0.2)	1.08 (0.16)	1.02 (0.16)	0.006
PTH	(1.6–6.9 pmol/l)	3.5 (3.12)	2.18 (0.86)	2.10 (1.10)	0.001
Magnesium	(0.71–0.94 mmol/l)	0.80 (0.07)	0.80 (0.06)	0.79 (0.08)	0.34
Alkaline phosphatase	(35–105 U/l)	129.13 (87)	105.00 (43)	107.00 (65)	0.30

ANOVA *p* value. Values are displayed as mean (SD).

**Table 4:** Sex hormones in males and females.

	Normal range	Overall	BMD within the normal range	Osteoporosis	*p* between groups
Males (*n* = 58)					
FSH	(<11 IU/l)	5.36 (3.07–9.18)	6.36 (3.09–10.62)	4.10 (2.96–5.45)	0.68
LH	(1.7–8.6 IU/l)	4.89 (3.79–6.74)	5.22 (3.96–7.32)	4.29 (2.69–6.02)	0.71
Testosterone	(8.6–29 nmol/l)	14.65 (10.47–17.79)	15.05 (11.32–18.13)	9.38 (9.02–12.07)	0.002
Estradiol	(0.09–0.22 nmol/l)	0.09 (09–.0.11)	0.09 (0.09–0.12)	0.09 (0.09–0.11)	0.40
Females (*n* = 55)					
FSH	(1.9–20 IU/l)	5.04 (3.77–6.58)	4.82 (3.77–6.70)	5.12 (3.69–6.29)	0.51
LH	(1.0–95.6 IU/l)	5.65 (2.65–8.81)	5.57 (2.61–8.92)	6.26 (2.10–7.97)	0.39
Testosterone	(0.4–1.7 nmol/l)	0.83 (0.47–1.39)	0.81 (0.42–1.35)	1.39 (0.61–3.21)	0.61
Estradiol	(0.05–1.46 nmol/l)	0.15 (0.11–0.33)	0.19 (0.11–0.35)	0.12 (0.09–0.13)	0.003

All data presented as median and interquartile range (IQR). FSH: follicle-stimulating hormone; LH: luteinizing hormone; n: nano; l: liter; IU: international units.

**Table 5:** Difference in baseline characteristics between osteoporotic and nonosteoporotic groups.

	BMD within the normal range (*N* = 106)	Osteoporosis (*N* = 19)	*p* between groups
ppFEV1 (IQR)	70 (52–87)	44 (27–65)	<0.001
BMI kg/m^2^ (IQR)	22.5 (20.4–24.5)	19.6 (18.8–20.7)	<0.001
Age, years (IQR)	30 (24–39)	34 (25–47)	0.52
Males, *n* (%)	55 (52)	7 (37)	0.27
Females, *n* (%)	51 (48)	12 (63)	—
Pancreas insufficiency, *n* (%)	101 (95)	19 (100)	ns
Homozygous F508del, *n* (%)	74 (70)	16 (84)	0.38
CF-related diabetes, *n* (%)	43 (39)	8 (53)	0.03
CF-related diabetes, duration in years (IQR)	9 (6–16)	15 (10–25)	0.06
Chronic infection, *n* (%)	82 (77)	19 (100)	0.02
Steroid inhalation therapy, *n* (%)	52 (47)	3 (20)	0.06
Additional D_3_ supplementation, *n* (%)	36 (34)	11 (58)	0.05
Proton pump inhibitors, *n* (%)	41 (39)	2 (16)	0.07

ppFEV1: percent predicted forced expiratory volume in 1 second; BMI: body mass index; additional D_3_ supplementation: additional D_3_ supplementation in addition to routine supplementation; BMD: bone mineral density; IQR: interquartile range.

**Table 6:** Univariate regression between spine and femoral *T* and *Z* scores and biochemical and clinical parameters.

	Spine *Z* score		Femoral *Z* score		Spine *T* score		Femoral *T* score	
	B	95% CI	B	95% CI	B	95% CI	B	95% CI
Albumin	0.03	(−0.01–0.07)	^∗^0.05	(0.01–0.09)	^∗^0.05	(0.002–0.09)	^∗^0.08	(0.03–0.12)
PTH	−0.52	(−1.31–0.27)	^∗^−0.19	(−1.59 to −0.02)	−0.69	(−1.46–0.28)	^∗^−0.23	(−0.36 to −0.09)
25OHD	0.001	(−0.01–0.01)	0.02	(−0.08–0.13)	0.04	(−0.07–0.16)	0.03	(−0.09–0.15)
Calcium	0.84	(−0.50–2.18)	^∗^1.36	(0.004–2.67)	1.41	(−0.06–2.88)	^∗^1.93	(0.41–3.47)
hbA1c	^∗^−0.25	(−0.44 to −0.05)	^∗^−0.38	(−0.57 to −0.18)	^∗^−0.35	(−0.56 to −0.13)	^∗^−0.46	(−0.69 to −0.26)
hsCRP	−0.36	(−0.75–0.32)	−0.62	(−1.01 to −0.23)	−0.38	(−0.81–0.05)	^∗^−0.69	(−1.14 to −0.25)
ppFEV1	^∗∗^0.02	(0.01–0.02)	^∗∗^0.03	(0.02–0.03)	^∗∗^0.02	(0.01–0.03)	^∗∗^0.03	(0.03–0.04)
BMI	0.05	(−0.01–0.11)	^∗^0.06	(0.003–0.12)	^∗∗^0.13	(0.07–0.2)	^∗∗^0.12	(0.05–0.20)

^∗^ *p* < 0.05; ^∗∗^*p* < 0.001.

**Table 7:** Multivariate Linear Regression for spine and femoral T and Z score.

Variables	B	SE of B	95% CI of B	*β*	B	SE of B	95% CI of B	*β*
*Femoral T score*					*Femoral Z score*			
Female gender	0.407	0.23	-0.045 - 0.859	0.15	0.349	0.14	-0.075 - 0.775	0.14
BMI	0.056	0.04	-0.012 - 0.125	0.15	0.012	0.03	-0.052 - 0.076	0.04
ppFEV1	^∗∗^0.027	0.01	0.017 - 0.038	0.46	^∗∗^0.022	0.01	0.012 - 0.032	0.42
Chronic Infection	^∗^-0.643	0.26	-1.166 to -0.120	-0.18	^∗^-0.617	0.25	-1.105 to -0.13	-0.20
Vitamin D	-0.017	0.05	-0.112 - 0.078	0.06	-0.011	0.05	-0.100 - 0.078	-0.02
Estradiol	-0.059	0.42	-0.891 - 0.773	-0.01	-0.110	0.40	-0.883 - 0.669	-0.02
HsCRP log10	-0.292	0.21	-0.705 - 0.12	-0.11	-0.30	-0.13	-0.681 - 0.089	-0.13
*Spine T score*					*Spine Z score*			
Female gender	^∗^0.598	0.23	0.133 – 1.062	0.23	^∗^0.560	0.22	0.115 – 1.003	0.24
BMI	^∗∗^0.108	0.04	0.037 – 0.178	0.29	0.027	0.03	-0.047 – 0.094	0.08
ppFEV1	^∗∗^0.015	0.01	0.004 – 0.026	0.26	^∗^0.012	0.01	0.002 – 0.023	0.24
Chronic Infection	^∗^-0.633	0.27	-1.170 to -0.096	-0.19	^∗∗^-0.724	0.30	-1.237 to –0.211	-0.24
Vitamin D	0.003	0.05	-0.094 – 0.101	0.01	0.000	0.06	-0.093 – 0.094	0.00
Estradiol	0.190	0.43	-0.664 – 1.044	0.04	0.18	0.41	-0.636 – 0.997	0.04
hsCRP log 10	-0.033	0.21	-0.457 – 0.391	-0.01	-0.046	0.20	-0.451 – 0.360	-0.02

^∗^ *p* < 0.05. ^∗∗^*p* < 0.01. Vitamin D is square rooted and hsCRP log10 transformed. Model fit: Femoral T score: *R*^2^ 0.39, adj. *R*^2^ 0.36, *F*(7/113)=10.71, *p* < 0.001. *Femoral Z score*: *R*^2^ 0.27, adj. *R*^2^ 0.32, *F*(7/113)=7.61, *p* < 0.001. *Spine T score*: *R*^2^ 0.31, adj. *R*^2^ 0.26, *F*(7/113)=7.15, *p* < 0.001. *Spine Z score*: *R*^2^ 0.22, adj. *R*^2^ 0.17, *F*(7/113)=4.58, *p* < 0.001.
